# Tensile strength of conventional glass ionomer cement and silver reinforced glass ionomer cement

**DOI:** 10.6026/973206300200391

**Published:** 2024-04-30

**Authors:** Suman Sharma, Anandamoy Bagchi, Shruti Mullick, Dubey Deepyanti, Katta Datta Sai Kiran, Amit Kumar, Pratik Surana

**Affiliations:** 1 Department of Pediatric Dentistry, Pihu Dental Hospital, Noida, Uttar Pradesh, India; 2 Department of Pedodontics & Preventive Dentistry, Kalinga Institute of Dental Sciences, KIIT Deemed to be University, Bhubaneswar, India; 3 Department of Prosthodontics Crown Bridge & Implantology, Sardar Patel Postgraduate institute of Dental and Medical Sciences, Lucknow, Uttar Pradesh, India; 4 Department of Conservative Dentistry and Endodontics, Dr D.Y Patil Dental College and Hospital, Dr D.Y Patil Vidyapeeth, Pimpri, Pune, Maharashtra, India; 5 Department of Conservative Dentistry and Endodontics, G. Pulla Reddy Dental College and Hospital, Kurnool, Andhra Pradesh, India; 6 Department of Dentistry, Lord Buddha Koshi Medical College and Hospital, Baijnathpur, Saharsa, Bihar-852221, India; 7 Department of Pedodontics and Preventive Dentistry, Maitri College of Dentistry and Research Centre, Durg, Chhattisgarh, India

**Keywords:** Glass ionomer cement, GIC, silver reinforced GIC, compressive strength

## Abstract

A comparative analysis and assessment of the compressive strength (CS) and diametral tensile strength (DTS) between conventional glass ionomer cement (C-GIC) and a silver-reinforced GIC (S-GIC) variant is of interest. Ten specimens of both C-GIC (GC Fuji II, Japan) and S-GIC (Riva Silver, SDI, Australia) were fabricated for the evaluation of compressive strength, and an identical number of samples were created for the examination of tensile strength. These specimens were then tested using a universal testing apparatus. The results exhibited that both the compressive and diametral tensile strengths were significantly greater for the S-GIC cement in comparison to the C-GIC, with a notable p-value of 0.001. The findings suggest that S-GIC may be considered a viable alternative to conventional GIC.

## Background:

Dental caries represents a prevalent chronic condition affecting individuals across all age groups on a global scale. In response to the advances in dental science, there has been a shift towards the development of novel, minimally invasive methodologies and materials aimed at preserving existing tooth structures and reducing the risk of injury to the dental pulp. [1, 2] Even after the meticulous removal of the carious layer through such conservative methods, there remains a possibility of residual infected tissues and microbial presence within the dentin cavity. [3, 4] This challenge has underscored the necessity for the innovation of materials that bolster demineralization and exhibit enhanced antibacterial effectiveness, primarily through their capacity for fluoride release. Among the materials championed for these purposes, Glass Ionomer Cements are notably recognized and were first introduced into the field of dentistry by Wilson and Kent in the 1970s. [5, 6] The advent of Glass Ionomer Cement (GIC) garnered global interest among dental professionals. Its characteristics such as adherence to moist tooth surfaces and base metals, anti-caries properties and minimal toxicity distinguish it as a distinct type of cement. Furthermore, its application simplicity, obviating the need for adhesive systems, enhances its clinical appeal. [7] Nevertheless, despite these advantages, traditional forms of GIC are impeded by several drawbacks, including brittleness, diminished fracture resistance, extended curing periods, and vulnerability to moisture and dehydration. [8] To ameliorate these deficiencies, progress has been achieved historically and persists through the integration of filler constituents in powder form, including silver particles, zirconia, and hydroxyapatite. Furthermore, liquids have been enhanced with additional polyacids, alongside the pre-treatment of the glass surface and alterations in glass compositions. [1] Riva Silver, a silver-reinforced GIC, aimed at enhancing the durability and performance of glass ionomer restoratives. Upon the introduction of novel materials, it is imperative to possess a comprehensive understanding of their physical and mechanical properties, as well as to conduct clinical evaluations, prior to their adoption in clinical settings. [9] The compressive strength test and the diametral tensile strength test emerge as the predominant methodologies for evaluating the mechanical characteristics of the newly introduced cements. Therefore, it is of interest to conduct a comparative analysis and evaluation of the CS and DTS between a traditional Glass Ionomer Cement and a Silver-reinforced GIC.

## Materials and Method:

The experimental design included the preparation of t specimens for each of the two materials; S-GIC (GC Fuji II) and S-GIC (Riva Silver, SDI), dedicated to both the CS and DTS evaluations, culminating in a total of ten samples. The dimensions for the CS testing cylinders were established at a diameter of 6.0 mm and a height of 12.0 mm, whereas for DTS testing, the dimensions were set at a diameter of 6.0 mm and a height of 3.0 mm, with all specimens encased in aluminum molds. The proportion of powder to liquid utilized in the preparation of these materials adhered strictly to the guidelines provided by the manufacturer. Subsequent to the mixing process, the resultant material was carefully transferred into plastic molds using a designated plastic instrument, followed by the application of acetate strips to each side of the mold. Thereafter, the assembly was placed within an incubator, maintained at a temperature of 37 ± 1°C and a relative humidity of 95 ± 5%, for duration of 1 hour to closely emulate oral environmental conditions. Upon removal from the molds, the pellets underwent a finishing process, being smoothed with 500-grit Silicon carbide paper. Subsequent analyses were conducted using a universal testing machine (Instron 1500 HDX), with the CS tests performed at a crosshead speed of 1 mm/min, and DTS tests at a speed of 0.5 mm/min. During the CS assessment, the cylindrical specimens were oriented to bear force longitudinally. Conversely, for the DTS evaluation, the specimens were subjected to diametral compression, thereby inducing a plane of tensile stress that corresponded with the force applied. Comparative analysis of CS and DTS values between the two groups of materials was conducted using an independent t-test for each parameter, setting a threshold for statistical significance at p ≤ 0.05.

## Result:

C-GIC and S-GIC had compressive strengths of 67 ± 12 and 121 ± 15 MPa, in that order. When compared to C-GIC, the compressive strength of S-GIC was noticeably higher (p < 0.001). Comparably, DTS also displayed a similar pattern for C-GIC, registering 10.8 ± 1.5 MPa, which was considerably less than that of S-GIC (28.4 ± 5.65 MPa). (Table 1)

## Discussion:

Glass Ionomer Cement stands as a significant material in dentistry, primarily due to its ability to chemically bond to tooth structure-both dentin and enamel. This uniqueness bestows it with considerable benefits, notably its biocompatibility and the capacity to release fluoride, which can aid in preventing further decay. However, despite its advantages, GIC is not without its drawbacks. It's relatively poor mechanical properties, including reduced strength and wears resistance; limit its application, particularly in posterior restorations where the bite forces are high. [2,3] In addressing these limitations, the dental materials research community has been engaged in developing enhanced forms of GIC.[1] These efforts aim to bolster the cement's physical properties without significantly compromising its inherent advantages. Among the innovations, the introduction of silver-reinforced GIC represents a notable advancement. Silver-reinforced GIC includes the incorporation of silver particles into the glass ionomer matrix. [8, 9] Result of resent In-vitro study showed a significantly higher CS and DTS of the S-GIC over the C-GIC underscores a critical advancement in dental material technology. The addition of silver particles is intended to increase the compressive and tensile strength of the cement. This makes it more capable of withstanding the forces exerted during chewing, especially in posterior areas of the mouth. By enhancing the material's resistance to wear, silver-reinforced GIC can better maintain its integrity and function over time, even in high-stress environments [10, 11]. However, while the results are promising, certain limitations must be acknowledged. The sample size, though adequate for initial exploration, is relatively small. A larger sample could provide a more robust statistical analysis and validate the findings across a broader spectrum of conditions. Additionally, in vitro studies, such as this one, do not entirely replicate the complex environment of the oral cavity. Factors such as saliva, temperature fluctuations, and microbial presence could affect the material properties differently, which warrants caution when extrapolating these results to clinical scenarios.

## Conclusion:

Data shows that silver-reinforced GIC can be used as alternative to conventional glass ionomer cement. The material of choice can be customized to meet specific needs by taking into account a number of variables, including cost-effectiveness, the surface that needs to be repaired, moisture contamination, and time constraints.

## Figures and Tables

**Table 1:** Mean CS and DTS Value of both samples

**Sample**	**Test**	**Mean Value ± Standard Deviation (MPa)**	**P-value**
Conventional GIV(C-GIC)		67 ± 12	
Silver Reinforced GIC (S-GIC)	CS	121 ± 15	0.001*
Conventional GIV (C-GIC)		10.8 ± 1.5	
Silver Reinforced GIC (S-GIC)	DTS	28.4 ± 5.65	0.001*
*= Significant
